# Statin-Associated Immune-Mediated Necrotising Myopathy With Troponin Elevation Without Cardiac Involvement in an Octogenarian

**DOI:** 10.7759/cureus.117532

**Published:** 2026-10-06

**Authors:** Arkar Myo, Ailie McKinty, William Kah Howe Lee, Alice Reid

**Affiliations:** 1 General Medicine, Royal Devon and Exeter Hospital, Exeter, GBR; 2 General Surgery, Bristol Royal Infirmary, Bristol, GBR

**Keywords:** creatine kinase, elderly people, lower limb weakness, prednisone treatment, statin-induced necrotizing autoimmune myopathy

## Abstract

Immune‑mediated necrotising myopathy (IMNM) is a rare autoimmune muscle disease characterised by progressive proximal weakness, markedly elevated muscle enzymes, and necrotising changes on biopsy with minimal inflammation. The anti‑HMGCR (anti-3-hydroxy-3-methylglutaryl coenzyme A reductase) subtype is strongly associated with statin exposure, and symptoms frequently persist despite discontinuation, often requiring immunosuppressive therapy. Although cardiac involvement is uncommon, the interpretation of raised troponin can be challenging, as regenerating skeletal muscle may express foetal troponin isoforms, creating diagnostic uncertainty. We report a rare case of statin‑associated IMNM in an octogenarian who developed progressive proximal weakness and sustained biochemical evidence of muscle injury after long‑term statin use. The persistence of her symptoms and enzyme elevation despite statin withdrawal, together with an unexpectedly raised troponin in the context of exertional breathlessness, prompted comprehensive evaluation to clarify the underlying cause and exclude alternative pathology. The subsequent identification of strongly positive anti‑HMGCR antibodies supported the diagnosis of statin‑associated IMNM, and she improved following immunosuppressive therapy. This case highlights the importance of recognising statin‑associated IMNM in older adults on statins presenting with persistent weakness and persistent elevated muscle enzymes. Awareness of the diagnostic pitfall, particularly in the coexistence of raised creatine kinase and troponin, along with early antibody testing and careful cardiac evaluation, can help avoid misinterpretation and facilitate timely treatment. The case reinforces the need for clinical vigilance when assessing unexplained weakness in later life and underscores the value of considering this condition even when initial investigations are inconclusive.

## Introduction

Immune‑mediated necrotising myopathy (IMNM) is a rare subtype of idiopathic inflammatory myopathy characterised by progressive proximal weakness, markedly elevated creatine kinase (CK), and muscle fibre necrosis with minimal inflammatory infiltrate [1-4]. Anti-3-hydroxy-3-methylglutaryl coenzyme A reductase (anti‑HMGCR) IMNM is most frequently associated with statin exposure, although it may also occur in statin-naïve individuals [2]. It most commonly affects individuals in the sixth and seventh decades of life, although cases have been reported across a broader age spectrum and differ from self‑limited statin myopathy by its persistence despite statin withdrawal and its need for immunosuppressive therapy, often with high‑dose corticosteroids and, in refractory cases, additional steroid‑sparing agents [1-4]. Although cardiac involvement is uncommon, interpretation of troponin elevation can be challenging because troponin release may arise from skeletal muscle injury or systemic inflammation rather than myocardial damage, necessitating careful diagnostic evaluation supported by imaging and antibody testing [5,6]. Awareness of these complexities is essential to avoid misdiagnosis and to ensure timely recognition of this uncommon but clinically significant condition.

This case was recently presented as a poster at ICNMD 2026 (International Congress on Neuromuscular Diseases 2026) between 7 July 2026 and 11 July 2026 in Florence, Italy.

## Case presentation

Clinical course

A woman in her early 80s first attended her general practitioner (GP) two weeks before hospital admission with a three‑month history of worsening proximal muscle weakness. Her GP stopped her atorvastatin 20 mg, which she had been taking daily for two to three years for dyslipidaemia, and arranged blood tests. These showed a markedly elevated CK level of 4,636 U/L. At her subsequent general practitioner's review, she reported no improvement in her weakness and described new exertional breathlessness. This prompted repeat blood tests, including troponin. Results demonstrated persistently raised CK levels (3,965 U/L) and an elevated troponin T level (263 ng/L). The GP was concerned about the possibility of an unrecognised myocardial infarction, and she was subsequently referred urgently to hospital for further assessment.

Upon assessment on hospital admission, the patient first noticed difficulty getting out of a chair and climbing stairs and later struggled with overhead activities and core strength. There were no symptoms of myalgia, skin rashes, sensory disturbance, systemic features, chest pain, or any autonomic symptoms. The patient was active, previously fully independent in all activities of daily living. Her past medical history included dyslipidaemia, as previously stated, and a previous B12 deficiency, which was within the normal range throughout this time. Neurological examination confirmed bilateral proximal weakness (hip flexion 2/5, shoulder abduction 3/5) with preserved distal strength, intact sensation, brisk reflexes, and no cutaneous changes. The patient was unable to sit up from lying without assistance, which was reported as very abnormal for her. There were no signs of heart failure on examination.

Investigations

Table 1 shows the laboratory findings on admission.

**Table 1:** Laboratory Findings on Admission

Test	Results	Reference Range
Creatine kinase (CK)	4,636 U/L (initial result with GP)	25–200 U/L
	3,965 U/L (repeat result with GP)	
	2,827 U/L (result on first day of admission)	
Troponin T (TnT)	263 ng/L	14 ng/L
N-terminal pro-brain natriuretic peptide (NT-Pro-BNP)	255 ng/L	< 450 ng/L for age > 75 years
Antinuclear antibody (ANA)	Weakly positive (1:80)	<1:40
Extractable nuclear antigen (ENA) profile	Negative	Negative
Thyroid‑stimulating hormone (TSH)	3.16 mIU/L	0.4–4.0 mIU/L
HIV serology	Negative	Negative
Lyme serology	Negative	Negative
C‑reactive protein (CRP)	2 mg/L	<3.0 mg/L
White cell count (WCC)	6200 cells per microlitre	4000–11000 cells per microlitre
Vitamin D	22 nmol/L	50–75 nmol/L

Anti-HMGCR antibody was sent on admission. Following 10 days, the result came back strongly positive at > 200 units/mL (normal value: < 20 units/mL).

The cardiac investigations showed sinus bradycardia on electrocardiogram (ECG) with no evidence of ischaemia (Figure 1A). Cardiac magnetic resonance imaging (cMRI) demonstrated normal left and right ventricular size and wall thickness, with no regional wall motion abnormality or myocarditis-related changes (Figures 1B, 1C). An echocardiogram (Echo) was also performed and was normal.

**Figure 1:**
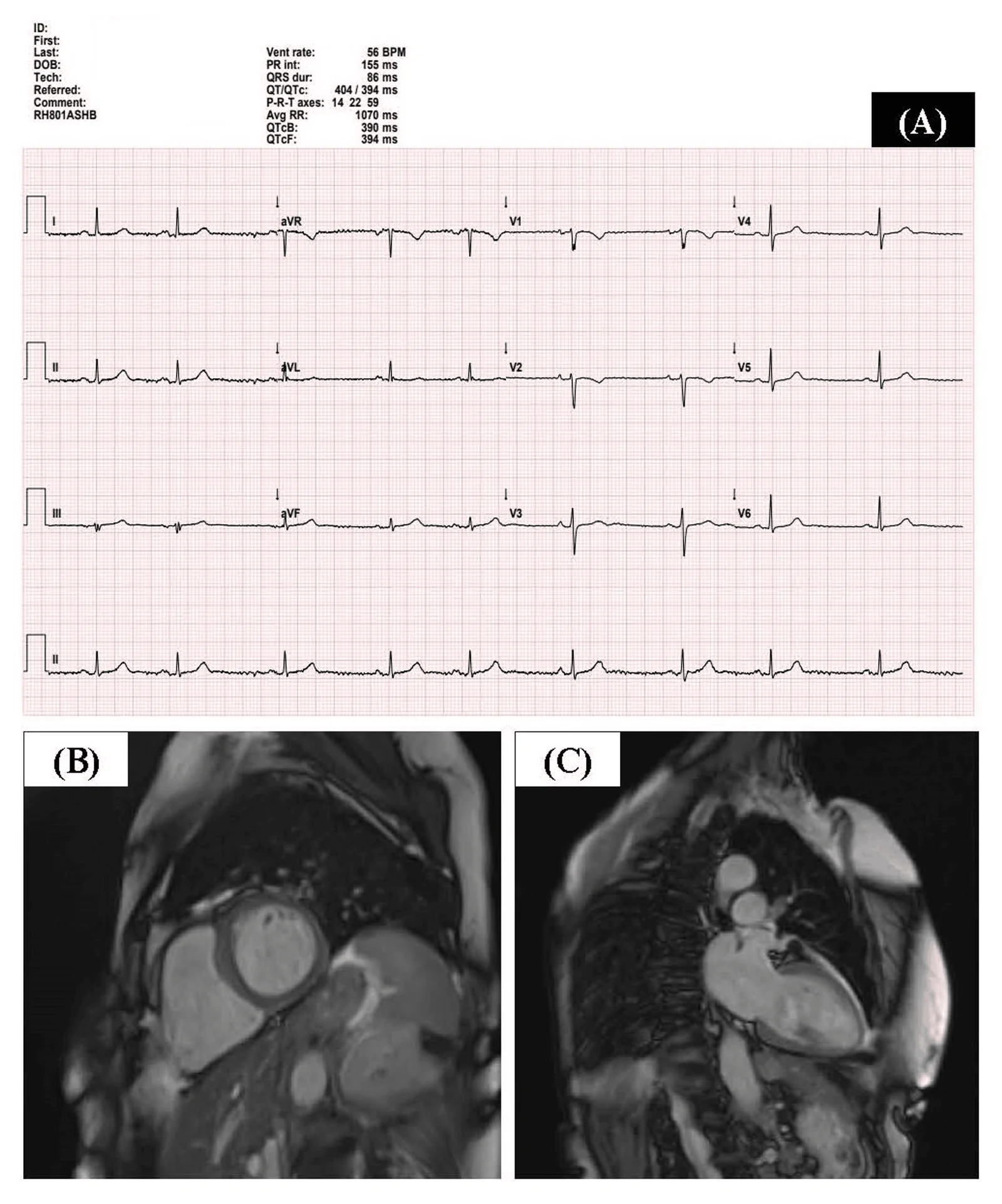
(A) ECG, (B) cMRI short-axis view, and (C) cMRI four-chamber view ECG showing sinus bradycardia with no evidence of acute ischaemic changes. cMRI short-axis view showing normal LV and RV size and wall thickness, with no regional wall motion abnormality or myocarditis-related changes. cMRI four-chamber view showing normal biventricular and biatrial chamber dimensions with no structural abnormality or no myocarditis-related changes. Abbreviations: ECG - electrocardiograph, cMRI - cardiac magnetic resonance imaging, LV - left ventricle, RV - right ventricle.

The computed tomography of the thorax, abdomen, and pelvis (CT TAP) (Figures 2A, 2B) and positron emission tomography (PET) (Figures 2C, 2E) were also performed, and the scans revealed no underlying malignancy.

**Figure 2:**
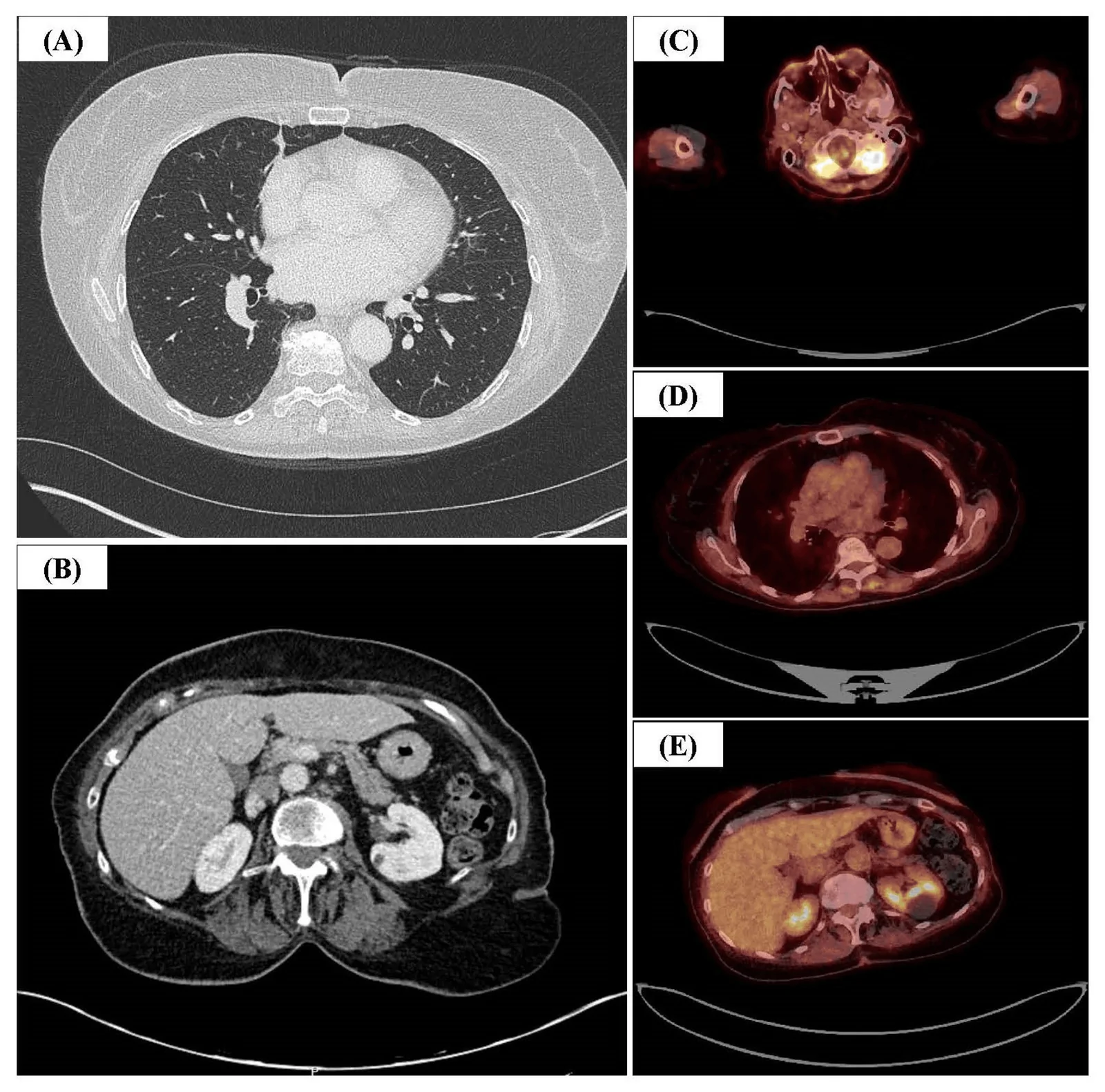
(A) CT thorax, (B) CT abdomen and pelvis, (C) PET head and neck, (D) PET thorax, and (E) PET abdomen and pelvis CT TAP and PET scan showing no evidence of underlying malignancy. Abbreviations: CT - computed tomography, PET - positron emission tomography, CT TAP - computed tomography thorax, abdomen and pelvis.

The muscle biopsy, performed in week six of steroid therapy as an outpatient at a tertiary centre, demonstrated occasional pale necrotic fibres with minimal inflammatory infiltrate, consistent with treated necrotising myopathy, as shown in the histopathological image below (Figure 3).

**Figure 3:**
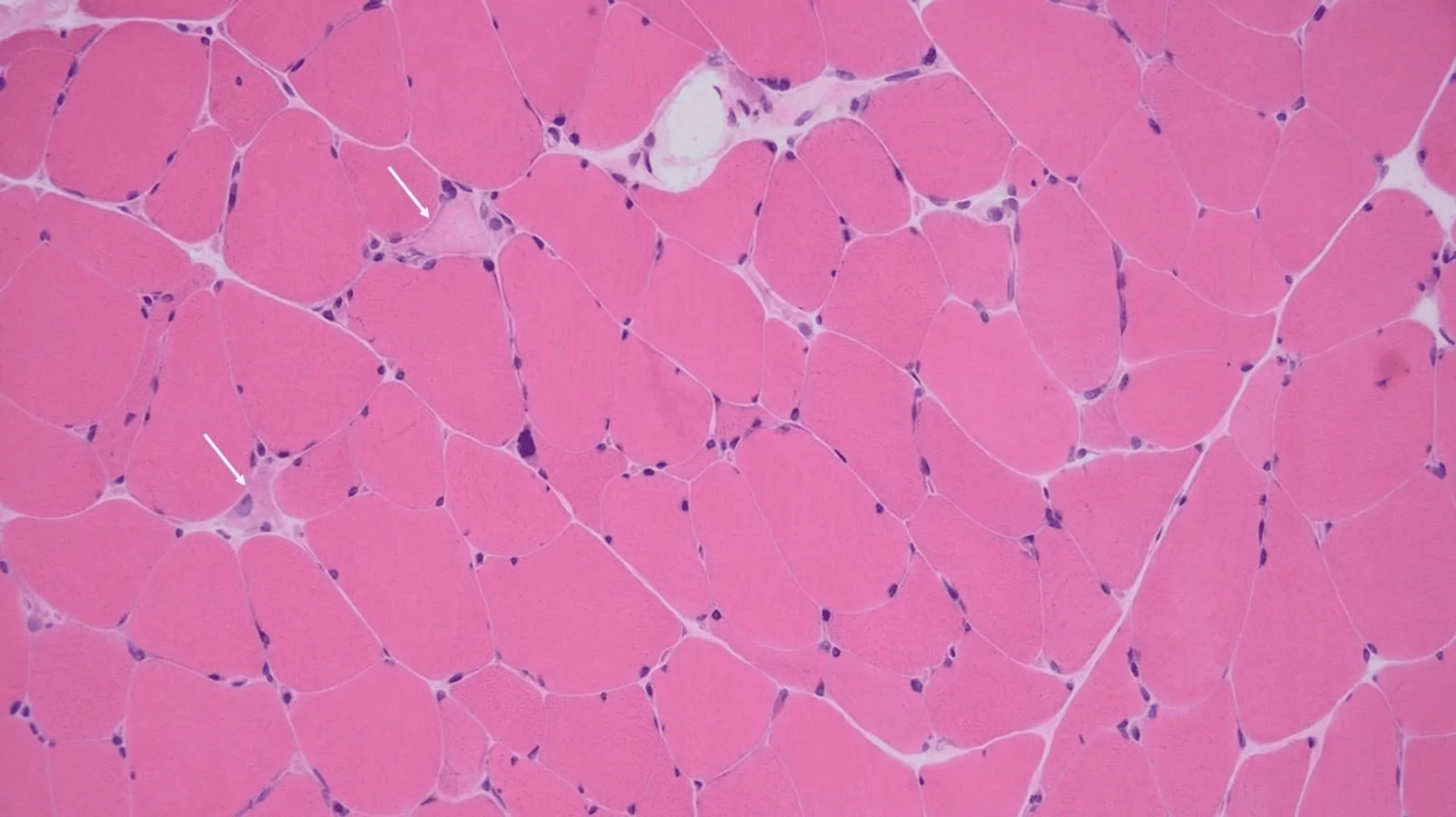
Micrograph of a muscle biopsy (right quadriceps) showing occasional pale necrotic fibres (white arrows) and lacking significant inflammation, suggestive of necrotising myopathy (H&E stain 200×)

Differential diagnoses

Several possibilities were considered at the presentation. Statin-induced toxic myopathy was initially suspected, given her history of atorvastatin use. However, her symptoms persisted despite discontinuation, and CK levels remained markedly elevated, making a purely toxic mechanism unlikely.

Inflammatory myopathies such as polymyositis, dermatomyositis, and inclusion body myositis, particularly considering the raised troponin, were also considered. However, the absence of cutaneous skin changes, a negative extended myositis antibody panel, and normal cardiac imaging made these less probable [1,4].

Paraneoplastic syndromes were explored given her age and subacute presentation, but computed tomography thorax-abdomen-pelvis and PET imaging revealed no evidence of malignancy.

Ultimately, the diagnosis of statin-associated IMNM was confirmed by a strongly positive anti-HMGCR antibody result (>200 units), which is highly specific for this condition [2,5]. Muscle biopsy findings supported the diagnosis and excluded other inflammatory or degenerative myopathies.

Treatment

Atorvastatin was initially withheld by the patient’s primary care two weeks prior to admission and was permanently stopped following the diagnosis. Once the anti-HMGCR antibody returned strongly positive (>200 units), high-dose oral prednisolone 40 mg daily was initiated, and she was discharged with rheumatology follow-up. After one week on prednisolone 40 mg daily, the dose was tapered by 5 mg weekly. She remained on maintenance prednisolone 5 mg daily from week seven of her steroid therapy onwards. Her CK level has increased to 2,272 U/L at week seven of steroid therapy despite excellent clinical improvement. The plan was made to continue her maintenance dose of prednisolone, as the specialist's impression was enzymes leaking, and to commence methotrexate if her repeat CK on the next follow-up did not continue to improve or kept rising. As her subsequent CK levels had improved, she continued to remain on maintenance prednisolone 5 mg daily with the aim to slowly wean under the rheumatology team.

Supportive care included physiotherapy to aid mobilisation, restore proximal strength, and improve safety at home. Given the vitamin D deficiency, a loading dose of vitamin D was administered, followed by maintenance therapy with combined calcium and vitamin D supplementation.

Outcome and follow-up

The patient had made steady progress following the initiation of steroid therapy. CK levels declined gradually, reflecting a good biochemical response to immunosuppression; however, there was an episode of rising CK levels to 2,272 U/L at week seven of steroid therapy despite good compliance with medication, without any history of intercurrent illness or overuse of muscles at that time. Fortunately, on subsequent blood tests, her CK levels continued to decrease, reaching 252 U/L at week 28 of steroid therapy. Given the subsequent improvement in CK levels, the isolated CK rise during an overall period of improvement was considered probably due to normal biological variability, laboratory measurement variation, enzyme leakage, or recent physical activity. Figure 4 depicts the trends in CK levels before and after the initiation of steroid therapy.

**Figure 4:**
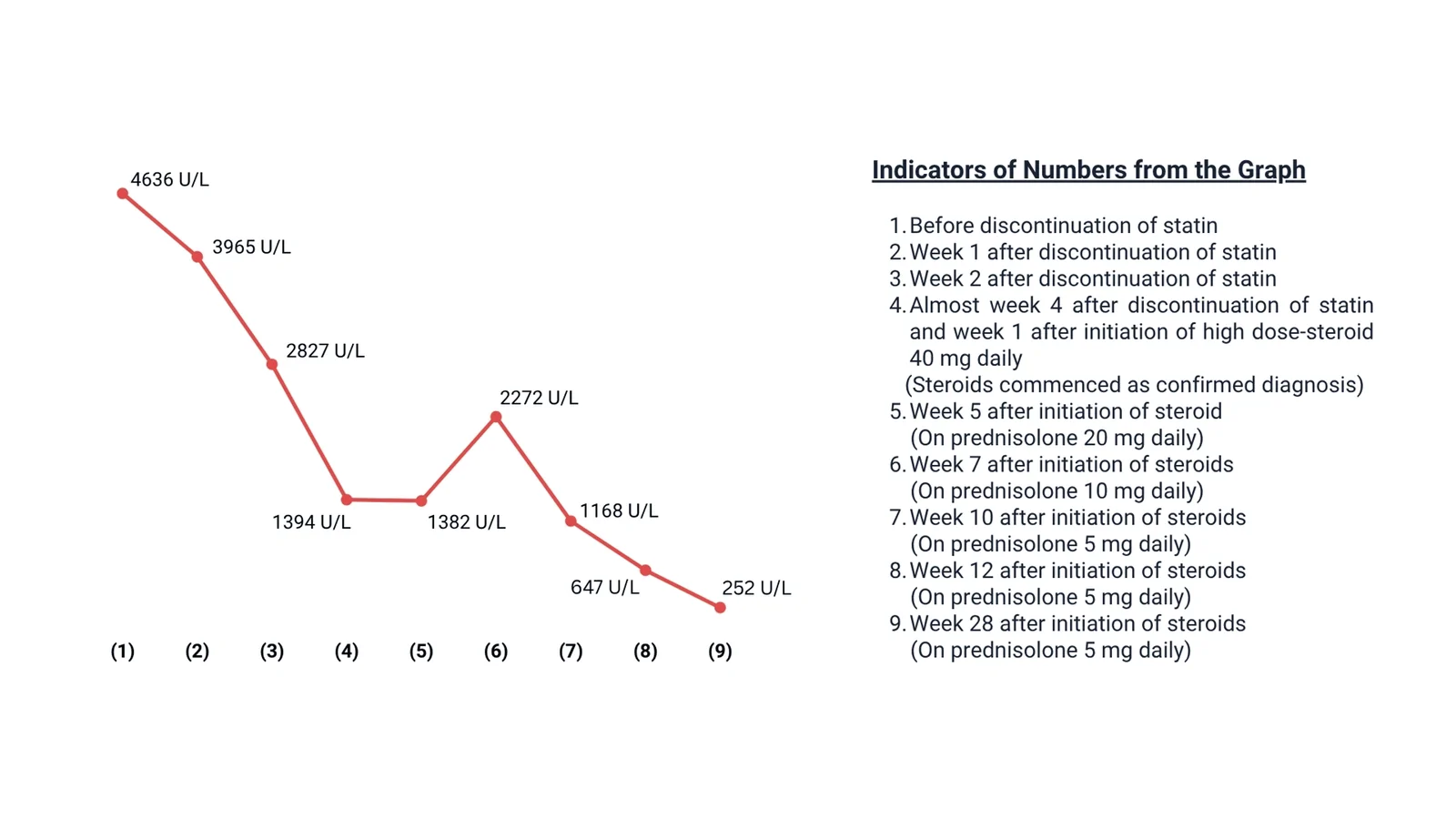
Trends of creatine kinase (CK) levels before and after initiation of steroid therapy

Cardiac markers also improved without the need for intervention, and imaging remained reassuring with no evidence of myocardial involvement.

Upon review at week 28 following steroid therapy, she achieved symptomatic remission with marked biochemical improvement and near-normalisation of CK levels. She remained functioning well independently with no issues in activities of daily living, and the plan was made to wean the prednisolone very slowly over four months and then stop. She remains to be reviewed regularly by the rheumatology team to monitor symptoms and assess the success of steroid weaning.

## Discussion

IMNM is characterised by proximal muscle weakness and elevated CK levels [3]. It is commonly divided into three subtypes based on the associated autoantibody: anti-signal recognition particle (anti-SRP) antibodies, anti-HMGCR antibodies, and seronegative cases (no identified antibody) [7]. Approximately two-thirds of IMNM cases have detectable autoantibodies [3].

In this case, anti-HMGCR antibodies were strongly positive. These antibodies are closely associated with statin-associated necrotising autoimmune myopathy, a rare immune-mediated process that leads to muscle necrosis [3,8]. The underlying muscle damage with associated elevated biochemical markers and ongoing symptoms despite statin withdrawal differentiates IMNM from myalgia, which is commonly seen with statin use [2].

HMGCR is a glycoprotein located on the endoplasmic reticulum (ER) and is essential for cholesterol synthesis; it is therefore a therapeutic target of statins [9]. The antibodies seen in anti-HMGCR IMNM target the same antigen as statins. Statins are thought to trigger IMNM in susceptible individuals by upregulating HMGCR expression and promoting autoimmune dysregulation, ultimately leading to muscle necrosis. The exact underlying mechanism, however, is not fully understood [2,3,9,10].

Some studies suggest that muscle damage results from activation of the complement pathway. This occurs through activation of the classical pathway, leading to sarcolemmal depositions and formation of transmembrane pores [7,11]. The resulting damage to muscle fibres leads to muscle necrosis through macrophage activation. This is in keeping with histology, which typically shows muscle necrosis with minimal inflammatory infiltrate and scattered regenerating fibres [4,10].

More recent evidence proposes that ER stress and autophagy may also contribute [7,9]. In this model, anti-HMGCR antibodies are internalised in the muscle and impair normal HMGCR function by disrupting intracellular metabolism. This triggers ER stress and activates autophagic pathways [9]. Although autophagy aims to support muscle regeneration, excessive or dysregulated activation can exacerbate muscle damage. While this view is not yet universally accepted, it may help explain why some patients do not respond to immunosuppressive treatments [9].

Myocarditis is rare in IMNM, and there was no evidence of cardiac involvement in this case. Elevated troponins, particularly troponin T, however, are often observed. While healthy adult skeletal muscle does not normally express troponin T (TnT), chronic muscle injury can lead to the re-expression of foetal muscle proteins, including TnT [5]. This phenomenon is seen in chronic myositis and other myopathies, where muscle damage can lead to TnT release into the bloodstream [5]. In this case, troponin isoform analysis was not performed because it was not available in routine clinical practice at our Trust. Furthermore, the presence of exertional breathlessness together with a markedly elevated troponin level warranted comprehensive cardiac evaluation irrespective of the potential contribution of regenerating skeletal muscle-derived TnT isoforms. This approach ultimately excluded clinically significant cardiac involvement. Regenerating skeletal muscle has been shown to re-express TnT isoforms, which may contribute to elevated circulating troponin concentrations in inflammatory and necrotising myopathies [5]. Although isoform analysis may provide additional mechanistic insight, it should not replace appropriate cardiac investigation when clinically indicated.

Several case series and reports have been published. These have shown that IMNM is more common in females and in Caucasian patients. On average, patients are in their 60s when they are diagnosed and have received prolonged courses of statin therapy, usually months to years [12]. Most patients present with proximal muscle weakness, but other symptoms include myalgia and dysphagia [3,12]. Patients often require a prolonged course of immunosuppressive treatment [1,3]. In around 60% of cases, remission is achieved, but a small number are refractory to treatment [12].

The aim of treatment for IMNM is to preserve and restore muscle function. It is important to promptly initiate immunosuppressive medication, as this has been shown to reduce the time until first remission [2,4,13]. As in this case, patients are commonly prescribed high-dose corticosteroids initially [2,3]. In many cases, steroids alone are not sufficient, and another agent is used. Alternative treatments include methotrexate, azathioprine, rituximab and intravenous immunoglobulin [4]. These therapies are useful in avoiding prolonged steroid use in patients who require longer courses of immunosuppression. In some patients, symptoms return soon after stopping treatment despite tapering doses. In these cases, ongoing low-dose immunosuppression may be required [1].

A limitation of this case is that the muscle biopsy was performed after six weeks of corticosteroid therapy. Immunosuppressive treatment may modify histopathological findings and reduce the diagnostic yield of muscle biopsy. Ideally, a biopsy should be obtained before treatment initiation whenever clinically feasible. However, in this case, the treatment was commenced because of the patient's progressive weakness, markedly elevated CK levels, and strongly positive anti-HMGCR antibodies, which are highly specific for anti-HMGCR IMNM [2,5], while biopsy arrangements at a tertiary centre were pending. Consequently, the biopsy findings should be interpreted in the context of prior corticosteroid exposure. Nevertheless, despite preceding corticosteroid therapy in this patient, the biopsy still demonstrated features consistent with necrotising myopathy, providing supportive histopathological evidence for the diagnosis.

## Conclusions

IMNM associated with statin exposure is an uncommon but clinically significant condition. Strongly positive anti‑HMGCR antibodies provide high diagnostic specificity and can streamline the diagnostic process, supporting earlier intervention and coordinated multidisciplinary follow‑up. Early recognition, timely antibody testing, and careful clinical oversight are central to achieving recovery and guiding safe corticosteroid tapering.

The coexistence of markedly elevated CK and raised troponin represents a cardinal diagnostic challenge, as skeletal muscle injury may contribute to troponin release and mimic cardiac involvement. Although cardiac troponin I (cTnI) measurement may help guide the evaluation of potential cardiac injury, it should complement rather than replace formal cardiac assessment. This case illustrates a rare presentation of statin-associated IMNM in an older adult with troponin elevation but no evidence of cardiac involvement on comprehensive cardiac assessment, emphasising that raised troponin in this context does not necessarily indicate myocarditis or other cardiac pathology. It reinforces the importance of systematic cardiac evaluation and thoughtful interpretation of troponin results in older adults, with consideration of troponin subtype measurement where clinically appropriate.
